# Detection of SARS-CoV-2 receptor binding domain using fluorescence probe and DNA flowers enabled by rolling circle amplification

**DOI:** 10.1007/s00604-023-05747-6

**Published:** 2023-03-29

**Authors:** Man Zhang, Lei Ye

**Affiliations:** Division of Pure and Applied Biochemistry, Department of Chemistry, Lund University, Box124, 22100 Lund, Sweden

**Keywords:** Rolling circle amplification, Fluorescence signal, DNA flower, Colorimetric detection, SARS-CoV-2 spike protein

## Abstract

Using rolling circle amplification (RCA) and two different ways of signal readout, we developed analytical methods to detect the receptor-binding domain (RBD) of SARS-CoV-2 spike protein (S protein). We modified streptavidin-coated magnetic beads with an aptamer of RBD through a biotin-tagged complementary DNA strand (biotin-cDNA). Binding of RBD caused the aptamer to dissociate from the biotin-cDNA, making the cDNA available to initiate RCA on the magnetic beads. Detection of RBD was achieved using a dual signal output. For fluorescence signaling, the RCA products were mixed with a dsDNA probe labeled with fluorophore and quencher. Hybridization of the RCA products caused the dsDNA to separate and to emit fluorescence (λ_ex_ = 488 nm, λ_em_ = 520 nm). To generate easily detectable UV–vis absorbance signal, the RCA amplification was extended to produce DNA flower to encapsulate horseradish peroxidase (HRP). The HRP-encapsulated DNA flower catalyzed a colorimetric reaction between H_2_O_2_ and 3,3′,5,5′-tetramethylbenzidine (TMB) to generate an optical signal (λ_abs_ = 450 nm). The fluorescence and colorimetric assays for RBD have low detection limits (0.11 pg mL^−1^ and 0.904 pg mL^−1^) and a wide linear range (0.001–100 ng mL^−1^). For detection of RBD in human saliva, the recovery was 93.0–100% for the fluorescence assay and 87.2–107% for the colorimetric assay. By combining fluorescence and colorimetric detection with RCA, detection of the target RBD in human saliva was achieved with high sensitivity and selectivity.

**Graphical Abstract:**

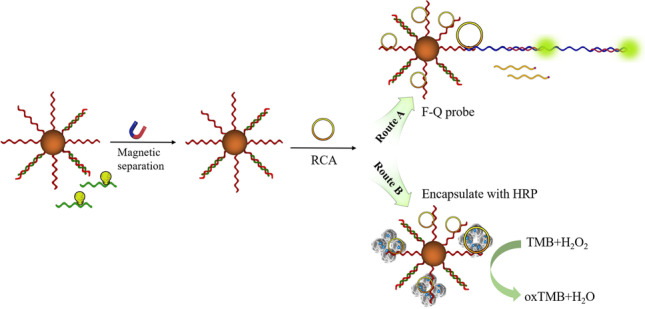

**Supplementary Information:**

The online version contains supplementary material available at 10.1007/s00604-023-05747-6.

## Introduction

Severe acute respiratory syndrome coronavirus 2 (SARS-CoV-2) is an enveloped virus with a surface heavily decorated with stick-like glycosylated spike (S) structural proteins, as well as three other major structural proteins: nucleocapsid (N), matrix (M), and envelope (E) proteins [1–3]. The structure of the S protein consists of three protomers, each containing S1 and S2 subunits, consisting of 672 and 588 amino acids, respectively [4, 5]. The S1 subunit is particularly important because it contains the receptor-binding domain (RBD) that plays a crucial role in SARS-CoV-2 virus infection. It is now well known that SARS-CoV-2 infects epithelial cells of the human respiratory system. The interaction between the RBD of the S protein and angiotensin-converting enzyme II (ACE2), which is expressed on the host cells, and the S2 subdomain are required for membrane fusion [6, 7]. In addition, the RBD determines the binding affinity to the host receptor, viral specificity, and mortality [8, 9]. For these reasons, the RBD of S protein provides a suitable target to develop biosensors for detection of SARS-CoV-2 [10].

There are many different tools to detect SARS-CoV-2 [11]. Some research groups have reported biosensors and molecular biology methods for SARS-CoV-2 detection by targeting RNA [12, 13], antigen and antibodies [14–16], and the spike and nucleocapsid proteins of the virus [17–19]. However, these assays have their own advantages and limitations. For example, RNA-based assays are rapid but can be affected by sample degradation, while antibody-based assays are highly specific but can be expensive. Nanomaterial-based biosensors have good specificity and can offer high-throughput, but reproducibility issues and potential toxicity exist. To overcome these limitations, we choose to develop aptamer-based biosensors for detection of SARS-CoV-2 RBD protein.

Aptamers have become a popular choice for constructing biosensors because of their high specificity and affinity for binding analytical targets. They offer advantages over antibodies [20], including unique 3D structure and variable conformation, stability across a wider temperature and pH range, and lower production costs [21–23]. Motivated by reported aptamers targeting the RBD of S protein [24], we decided to use aptamer as a recognition element to develop protein assays using isothermal amplification to achieve high sensitivity. Several isothermal amplification methods, for example, hybrid chain reaction (HCR) [25, 26] and rolling circle amplification (RCA) [27–30], are suitable for in situ signal amplification on the surface of solid materials. As DNA-encoded information is stable and can be converted easily into optical or electrochemical signals, DNA-based probes are routinely used in bioanalytical assays. The nucleic acid sequences can also be designed to form various interesting structures for signal output [31, 32].

The aim of this work is to develop analytical method for detection of SARS-CoV-2 RBD by fluorometric and colorimetric measurements. The optical assays are based on displacement of aptamer from its cDNA by the target RBD. A rolling circle amplification on magnetic beads is used to enhance the target-induced signal output. Two alternative means are considered for signal readout. For fluorometric reading, the RCA product is used to trigger fluorescence emission after hybridization with a dsDNA labeled with fluorophore and quencher. For colorimetric reading, the RCA is extended to produce DNA flower [33] to encapsulate horseradish peroxidase (HRP) [34, 35], which catalyzes the reaction between H_2_O_2_ and 3,3′,5,5′-tetramethylbenzidine (TMB) to alter the UV–vis absorbance. Using SARS-CoV-2 RBD as a model analytical target, we wish to highlight the potential of aptamers and RCA in biosensing applications.

## Experimental

### Reagents and apparatus

All HPLC-purified oligonucleotide sequences were synthesized by Sangon Biotech (Shanghai, China). The nucleotide sequences used in this work are given in Table S1. We designed all the DNA sequences expect for the aptamer. The buffer composition used in this study are listed in Table S2. Recombinant SARS-CoV-2 receptor-binding domain (RBD, source: HEK293-derived SARS-CoV-2 Spike RBD protein (Arg319-Phe541), with a 6x-His tag at C-terminal), recombinant MERS-CoV Spike RBD (Chinese Hamster Ovary-derived MERS-CoV Spike RBD protein (Glu367-Tyr606), with a 6x-His tag at C-terminal), recombinant SARS-CoV-2 B.1.617.2 RBD (source: HEK293-derived SARS-CoV-2 Spike RBD protein (Arg319-Phe541 (Leu452Arg, Thr478Lys), with a 6x-His tag at C-terminal), and recombinant SARS-CoV-2 nucleocapsid (source: *Spodoptera frugiperda*, Sf 21 (baculovirus)-derived SARS-CoV-2 nucleocapsid (Met1-Ala419), with a 6x-His tag at C-terminal) were purchased from Bio-Techne (Minneapolis, USA). Human saliva was purchased from Innovative Research, Inc. (USA). T-4 DNA ligase, 10 × T-4 DNA ligase buffer, Exo I and Exo III enzyme, phi29 DNA polymerase, NEBuffer 1, and dNTPs were obtained from New England BioLabs (Ipswich, MA, USA). Dynabeads™ M-280 streptavidin and hydrogen peroxide (H_2_O_2_) were purchased from Thermo Fisher Scientific Co., Ltd. (USA). Horseradish peroxidase (HRP) and 3,3′,5,5′-tetramethylbenzidine (TMB) were purchased from Sigma-Aldrich (Sweden). All the other chemicals (analytical reagents) were obtained from commercial sources and were used without additional purification. All buffers were prepared with purified water from a Milli-Q water purification system (Millipore Corp., Bedford, USA). Fluorescence measurements were performed with Cary Eclipse Fluorescence Spectrophotometer (Agilent Technologies, Germany). UV–vis spectrometry was carried out using a Cary 60 UV–vis spectrophotometer (Agilent Technologies, USA). SEM was carried out using JEOL JSM 6700F scanning electron microscope (Japan).

Preparation of cDNA/aptamer-functionalized magnetic beads (MBs-cDNA/apt) and synthesis of circular DNA template are described in the Electronic Supplementary Material (ESM).

### Competition experiment

S protein RBD solution was diluted to 0, 0.001, 0.01, 0.1, 1, 10, and 100 ng/mL in 1 × PBS buffer. The prepared solution (100 μL) was mixed with 50 μL of MBs-cDNA/apt suspension. The mixture was shaken at room temperature for 1 h. During this period, S protein RBD bound to the aptamer to cause it to dissociate from the cDNA immobilized on the MBs. After magnetic separation, the MBs were collected and washed 3 times with 200 μL 1 × PBS buffer. Finally, the MBs were collected and re-suspended in 30 μL 1 × PBS buffer to give MBs-cDNA to be used in RCA.

### Rolling circle amplification

Circular DNA template (2 μL) and MBs-cDNA (4 μL) were mixed with 1 μL recombinant albumin, 1-μL phi29 DNA polymerase (10 U/μL), 4-μL dNTPs (10 mM), 3 μL 10 × phi29 DNA polymerase buffer, and 15 μL H_2_O. The mixture was gently stirred and incubated at 30 °C. In the fluorometric assay, the RCA continued for 2 h before the mixture was heated at 65 °C for 10 min to inactivate the enzyme. In the colorimetric assay, 5-μL HRP solution (1 mg/mL) was added before the RCA was started and continued for 20 h. The last step of enzyme inactivation was omitted in the colorimetric assay.

### Gel electrophoresis

Non-native polyacrylamide gel (12%) was prepared using 3 mL 5 × TBE buffer, 6-mL water and 6 mL 30% acrylamide/bis-acrylamide (29/1), 10 μL TEMED, and 110 μL of 10% freshly prepared ammonium persulfate (APS) solution. Electrophoresis was run at 225 V for 35 min in 0.5 × TBE buffer. The gel was stained with Gel-green for 40 min and was visualized using a Bio-Rad gel imager.

### Fluorometric and colorimetric detection of RCA products

#### Fluorometric detection

Two DNA sequences were designed to construct the probe: one was modified with BHQ-1 at the 3′ terminal and the other one with FAM at the 5′ terminal. The two DNA solutions (10-μM stock solution) were mixed at room temperature for 1 h to prepare the F-Q probe. For detection of RCA products, 10 μL of the F-Q probe was added into 30 μL of amplification products and 60 μL H_2_O. The mixture was kept at room temperature for 45 min. For fluorescence measurement, the excitation wavelength was set to 488 nm, and the emission wavelength of the fluorescence peak was 520 nm.

#### Colorimetric detection

The RCA was extended to 20 h to synthesize the HRP-loaded DNA flowers. The DNA flowers were collected by magnetic separation, then washed with 100-μL water to remove excess HRP. Finally, the DNA flower was re-suspended in 30-μL water. For colorimetric detection, the DNA flower (10 μL) was mixed with 80-μL acetate buffer (pH 5, 0.1 M), then added to 5 μL 35% H_2_O_2_ (w/w aqueous solution) and 5-μL TMB (0.025 mM). The mixture was put on a rocking table at room temperature for 10 min. After adding 50 μL of stop solution (0.2 M H_2_SO_4_) into the mixture, the particles were removed by magnetic separation. The absorbance of the supernatant at 450 nm was measured using a UV–vis spectrophotometer. All data were obtained from triplicate experiments.

### Characterization of DNA flowers

DNA flowers were characterized by scanning electron microscopy (SEM). Prior to SEM imaging, the samples (10 μL) were dried in an oven and coated with palladium/gold to increase conductivity. The DNA flowers were characterized using excitation voltage of 3 ~ 10 kV.

### Detection of S protein RBD in saliva

Human saliva samples (100 μL) were analyzed directly using the fluorometric and colorimetric assays without pre-treatment. Saliva (100 μL) was mixed with 100 μL of S protein RBD solution (1–100 ng/mL). The spiked samples were used to measure the RBD concentration with the fluorometric and colorimetric assays. All the measurements were repeated in triplicate.

## Results and discussion

### Principle of fluorometric and colorimetric detection based on rolling circle amplification

**Scheme 1:**
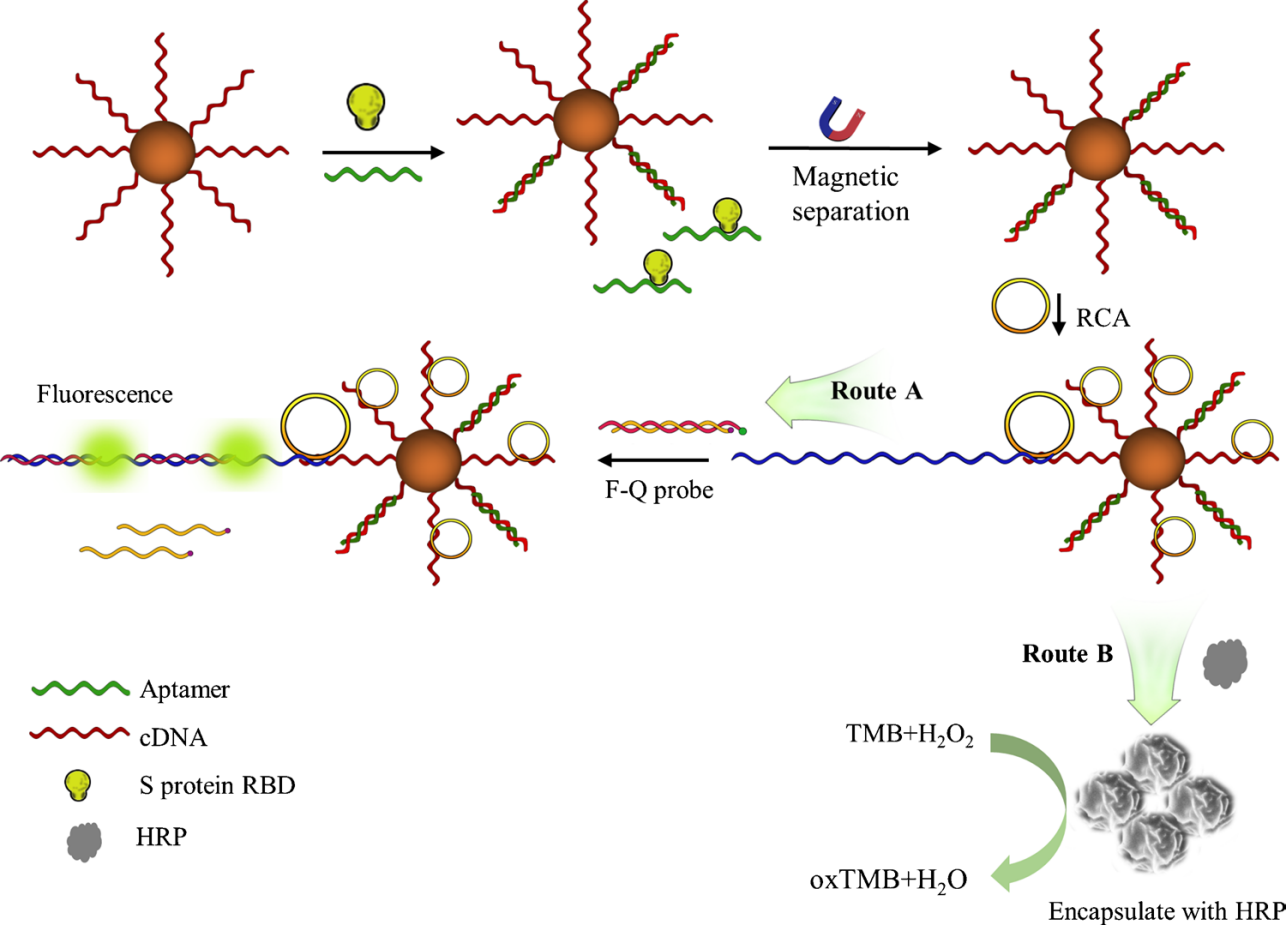
Principle of detection of SARS-CoV-2 spike protein RBD using fluorescence probe and DNA flowers based on rolling circle amplification

The principle of analyte detection is presented in Scheme 1. In this work, we used streptavidin-coated magnetic beads to immobilize biotin-cDNA/aptamer to form MBs-cDNA/aptamer. In the presence of S protein RBD, there is a competition between RBD and the cDNA to bind the aptamer. After a magnetic separation, the solution including the aptamer-S protein RBD can be removed. Therefore, there is a positive correlation between the concentration of S protein RBD and the un-complexed cDNA on the MBs. The un-complexed cDNA acts as a primer to trigger RCA after addition of the circular DNA template and phi29 DNA polymerase. After the polymerization, the first signal readout is achieved using the F-Q probe that hybridizes with the RCA products (RCAPs). Because of the complimentary sequences between RCAPs and the F probe, strand displacement occurs to eliminate the quenching caused by the Q probe. The second signal readout is achieved by increasing the reaction time of RCA to form HRP-loaded DNA flower. After magnetic separation and washing, the HRP loaded in the DNA flower is used to catalyze the colorimetric reaction between TMB and H_2_O_2_ to generate UV–visible signal.

### Rolling circle amplification

**Fig. 1:**
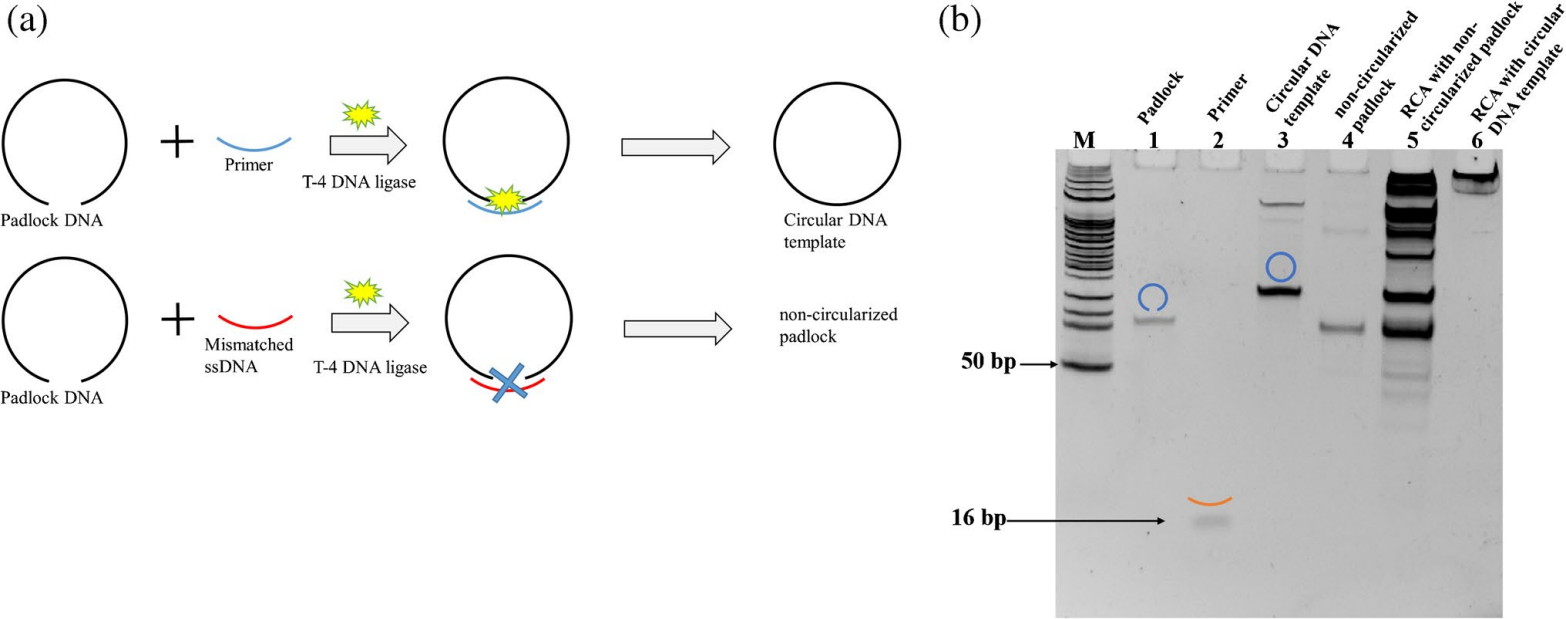
Rolling circle amplification. **a** Schematic illustration of synthesis of circular DNA template from padlock DNA using primer and T-4 DNA ligase. **b** Verification of the formation of circular DNA template and RCA by gel electrophoresis. M: 50 bp marker; padlock DNA (stock solution 1 μM); primer (stock solution 1 μM)

The importance of primer to match the padlock DNA for synthesis of the circular DNA template was also investigated. In Fig. 1a, the steps for synthesis of the circular DNA template are illustrated. A circularized DNA template can be formed only when the primer is fully complementary to pair with the padlock DNA. A mismatched ssDNA is not able to allow the T-4 DNA ligase to produce the circularized DNA template from the padlock DNA. Figure 1b shows the electrophoretic DNA bands from the padlock DNA, the primer, the circular DNA template, and the products of the rolling circle amplification using the circular DNA template. From the electrophoresis image, it is clear that the circular DNA template (lane 3) and the non-circularized padlock DNA (lane 4) display different mobility. The padlock DNA band in lane 1 and the non-circularized padlock DNA band in lane 4 have almost the same mobility, suggesting that the mismatched ssDNA was not able to give useful circular DNA template. In lane 3 the circular DNA structure has a slower mobility than the padlock DNA (lane 1). Lanes 5 and 6 are the products obtained by RCA using the non-circularized padlock DNA and circular DNA template. Compared to RCA using the non-circularized padlock DNA that produced heterogeneous products (lane 5), the circular DNA template produced only high molecular weight products represented by an intense band on the top of the gel image (lane 6).

### Fluorometric detection

**Fig. 2:**
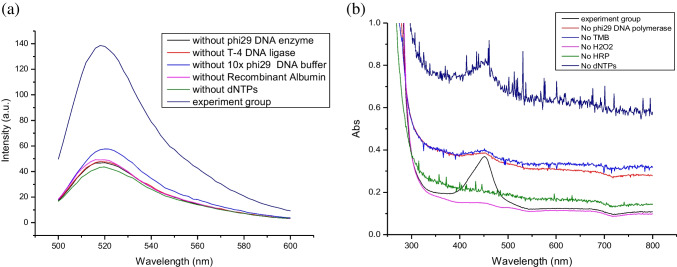
Feasibility study of the proposed fluorometric and calorimetric assays. **a** Fluorescence emission spectra of the final particle suspensions in the fluorometric assay. RBD concentration: 1 ng/mL. **b** UV–vis spectra of the final liquid phases in the calorimetric assay. RBD concentration: 1 ng/mL

To further prove the successful RCA-enabled fluorometric detection, we performed a series of fluorescence detection experiments by omitting one essential component of the RCA reaction. The results are compared with the RCA-enabled fluorescence detection (Fig. 2a). In contrast to the RCA-enabled fluorescence detection that gives a strong emission, when a single RCA reaction component (phi29 DNA polymerase, T-4 DNA ligase, dNTPs, or 10 × phi29 DNA polymerase buffer) is omitted, the fluorescence signal became significantly reduced.

### Colorimetric detection

To realize colorimetric detection for RBD, we selected to use HRP-loaded DNA flower to catalyze the commonly used colorimetric reaction between TMB and H_2_O_2_. As shown in Fig. 2b, when one of the essential components of the RCA reaction was missing, it was not possible to obtain a clear absorbance band as a response to RBD. Also, omitting any component of the colorimetric reaction (HRP, TMB, or H_2_O_2_) made it impossible to observe the absorbance band at 450 nm. These results confirm that both the RCA-enabled DNA flower and the HRP-catalyzed reaction are indispensable for the colorimetric detection of RBD.

**Fig. 3:**
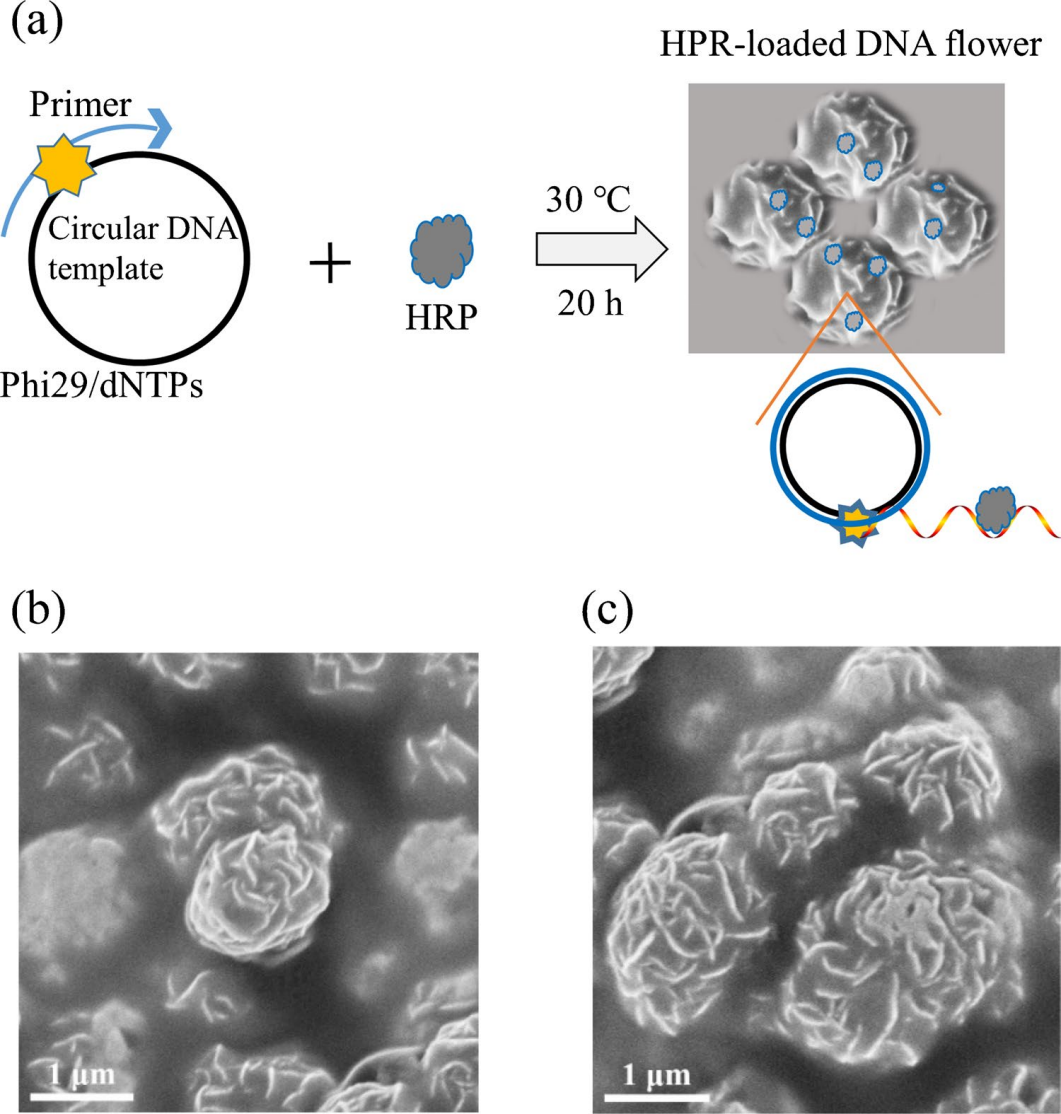
Formation of HRP-loaded DNA flowers by RCA. **a** Schematic of encapsulation of HRP in DNA flowers via rolling circle amplification. **b** SEM image of DNA flowers. **c** SEM image of HRP-loaded DNA flowers

Formation of HRP-loaded DNA flower can be achieved using the same RCA reaction as used in the fluorometric detection (Fig. 3a). The morphology of the DNA flowers (DFs), with and without loaded HRP, was studied by scanning electron microscopy (SEM) (Figs. 3b, c). The extended rolling circle amplification generated long DNA strands, which complexed with Mg_2_PPi to form flower-like microparticles. The uneven, petal-like structure of the DFs provides a larger surface area, making the encapsulated HRP readily accessible to the substrates TMB and H_2_O_2_. For colorimetric detection, the signal output depends on the amount of HRP trapped in the DNA flowers, as well as the concentration of TMB and H_2_O_2_.

### Evaluation of fluorometric and colorimetric methods for RBD detection

**Fig. 4:**
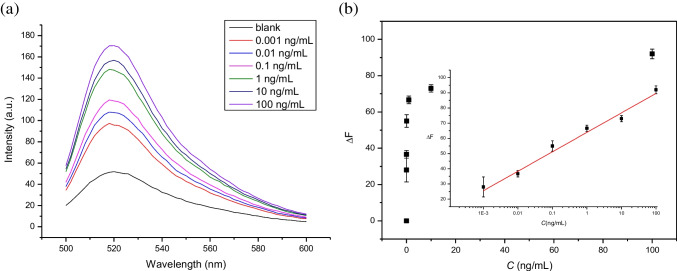
Fluorometric assay of S protein RBD enabled by RCA. **a** Fluorescence emission spectra of the final particle suspensions corresponding to different concentrations of S protein RBD (0.001–100 ng mL^−1^). **b** Relationship between the S protein RBD concentration and the fluorescence intensity change. For RBD concentration in the range of 0.001–100 ng mL^−1^, the linear equation is *y* = 12.8 *lg*(*x*) + 63.98 (with *R*.^2^ = 0.9703)

Using the optimized conditions, we studied the dose–response curves of the fluorometric and colorimetric methods for detection and quantification of S protein RBD. Standard solution of RBD of different concentrations (0, 0.001, 0.01, 0.1, 1, 10, 100 ng mL^−1^) was added to MBs-cDNA/apt. Complexation with RBD caused the aptamer to dissociate from the cDNA immobilized on the MBs. The un-complexed cDNA became available to trigger the RCA reaction. Figure 4a shows the change of fluorescence intensity in response to the varying RBD concentration. The correlation between RBD concentration and change of fluorescence intensity can be clearly seen in Fig. 4b. The semi-logarithmic plot shows a linear relation between fluorescence intensity and the logarithm of RBD concentration in the range of 0.001–100 ng mL^−1^, with a detection limit of 0.11 pg mL^−1^ based on 3σ/m criterion. For the fluorometric method, the total analysis time is ~ 4 h.

**Fig. 5:**
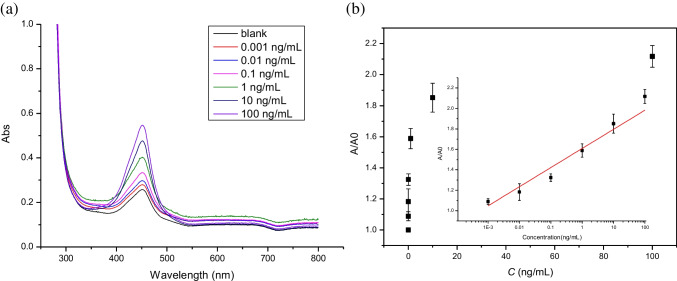
Colorimetric assay of S protein RBD enabled by RCA. **a** UV–vis spectra of the final liquid phases corresponding to different concentrations of S protein RBD (0.001–100 ng mL^−1^). **b** Relationship between the S protein RBD concentration and the Abs value. For RBD concentration in the range of 0.001–100 ng mL^−1^, the linear equation is *y* = 0.187 *lg*(*x*) + 1.61 (with *R*.^2^ = 0.9322)

Using the colorimetric method, we obtained a similar calibration curve for detection and quantification of RBD. As shown in Fig. 5, the absorbance of the assay solution increased linearly with increasing RBD concentration, in the semi-logarithmic plot in the range of 0.001 to 100 ng mL^−1^. The limit of detection of the colorimetric assay is 0.904 pg mL^−1^. For the colorimetric method, although the DNA flower synthesis takes 20 h, the signal readout time is very short (about 10 min).

**Fig. 6:**
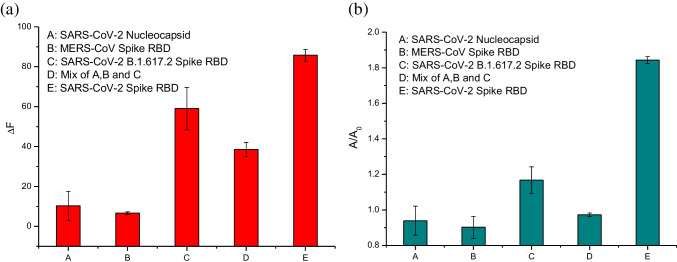
Evaluation of specificity of the RCA-enabled fluorometric (**a**) and colorimetric assay (**b**). The signal intensity generated from S protein RBD is compared with that from N protein, MERS protein RBD, SARS-Cov-2 B.1.617.2 spike protein RBD, and a mixture of the three proteins. The total protein concentration in each sample was 50 ng/mL

To evaluate the selectivity of the two analytical methods, we replaced RBD with a series of SARS-related proteins and carried out the protein assays. As shown in Fig. 6, the fluorescence and absorbance signals caused by all the other proteins are much weaker than SARS-CoV-2 RBD. Obviously, the selectivity of the present protein assays is due to the selective molecular recognition (RBD binding) provided by the aptamer. The RCA provides effective amplification of the molecular binding signal, making it possible to detect RBD at a very low concentration. A comparison of analytical performance between the present work and previously reported methods is provided in Table S3.

### Detection of S protein RBD in biological samples

The accuracy of the fluorometric and colorimetric assays was assessed by studying analyte recovery from spiked biological samples. Saliva samples were spiked with S protein RBD and analyzed. The assay protocols for determination of RBD are described in the Electronic Supplementary Material (ESM).

As shown in Table 1, the average recoveries of RBD from the spiked saliva samples were 93–100% and 87.2–107% for fluorometric and colorimetric assays, respectively. The relative standard deviation (RSD) of measurements were 4.03–10.6% and 6.15–9.04%, respectively. Therefore, the RCA-enabled protein assays based on fluorescence and absorbance measurements allow to detect and quantify SARS-CoV-2 RBD in untreated biological samples.

**Table 1:** Recovery of S protein RBD at different concentration levels in human saliva with two signal output methods (*n* = 6)

Methods	Spiked concentration in sample (ng/mL)	Standard deviation	Average-detected concentration (ng/mL)	Recovery (%)	RSD (%)
F-Q probe	0.5 5 50	9.89 8.37 4.04	0.465 4.95 50.1	93.0 98.9 100	10.6 8.45 4.03
DFs-HRP	0.5 5 50	7.88 6.15 8.90	0.436 5.00 53.5	87.2 100 107	9.04 6.15 8.27

For more complex samples such as blood and serum, optical detection in the visible wavelength range may be affected by some interfering substances. This limitation can be mitigated by using alternative labels, e.g., long wavelength fluorescent dyes or upconversion fluorophores.

## Conclusions

This work developed competitive fluorometric and colorimetric methods for detecting the receptor-binding domain protein of SARS-CoV-2 using aptamer and rolling circle amplification. The signal readout can be performed using fluorometric or colorimetric measurements, both of which offer accurate and reliable results. The RCA-enhanced methods have potential for virus particle detection. The principles of these protein assays can also be adapted for detecting other low-abundance proteins and biomarkers by changing the aptamer. One limitation of the present assays is the long analysis time, in particular the colorimetric method involving DNA flowers. This issue will be addressed in future studies using, e.g., faster signal amplification reactions.

## Supplementary Information

Below is the link to the electronic supplementary material.

Supplementary file1 (PDF 203 KB)

## Data Availability

All data generated or analysed during this study are included in this published article and its supplementary information files.
