# Supplementary material for: Detection of SARS-CoV-2 receptor binding domain using fluorescence probe and DNA flowers enabled by rolling circle amplification
Source: Mikrochim Acta. 2023 Mar 29;190(4):163. doi: 10.1007/s00604-023-05747-6 (PMC10052277; doi:10.1007/s00604-023-05747-6)
Supplement: Supplementary file 1 — Supplementary file1 (PDF 203 KB) [file 604_2023_5747_MOESM1_ESM.pdf]

Electronic Supplementary Material

Detection of SARS-CoV-2 receptor binding domain using fluorescence probe and DNA flowers enabled by rolling circle amplification

Man Zhang, Lei Ye*

Division of Pure and Applied Biochemistry, Department of Chemistry, Lund University, Box124, 22100 Lund, Sweden

***Corresponding authors: Lei Ye, E-mail: lei.ye@tbiokem.lth.se**

Preparation of cDNA/aptamer-functionalized magnetic beads

For the cDNA/aptamer-functionalized magnetic beads synthesis, we followed the method with a few modifications [1]. Aptamer stock solution (10 μM, 50 μL) was heated at 80 °C for 3 min. Biotin-cDNA stock solution (10 μM, 25 μL) was heated at 40 °C for 10 min. After cooling to room temperature in 1 h, the two solutions were combined and mixed with 125 μL of 1 × phosphate buffered saline (PBS). The obtained solution (aptamer/cDNA) was incubated at room temperature for 1 h. Streptavidin-modified magnetic beads (MBs) (200 μL, 10 mg/mL) were transferred into a low adsorption centrifuge tube. After magnetic separation and removing the solvent, the MBs were washed 3 × with 400 μL of binding buffer. The MBs were mixed with the solution of aptamer/cDNA and incubated at room temperature for 30 min. The particle suspension was then placed in a DynaMag magnet (ThermoFisher) for 3 min to separate the MBs. The MBs were washed three times with 1 × PBS buffer. After this step the MBs were treated with 200 μL freshly prepared 1% BSA at room temperature for 1 h. The MBs were separated and re-suspended in 600 μL 1 × PBS buffer. The aptamer/cDNA-functionalized magnetic beads (MBs-cDNA/apt) obtained were kept at 4 °C until use.

Synthesis of circular DNA template

We followed the method to synthesis circular DNA template with a few modifications [2]. Padlock DNA (10 μM, 2 μL), primer DNA (10 μM, 4 μL) and 1 μL of 10 × T-4 DNA ligase buffer were mixed and incubated at 95 °C for 5 min, then gradually cooled to room temperature (in 2 h). Subsequently, 1 μL T-4 DNA ligase (400 U/μL) and 2 μL H_2O were added. The mixture was incubated at room temperature for 2 h, and then at 65 °C for 10 min to inactivate the enzyme. The excess primer and padlock DNA were digested by adding 2 μL Exo I (20 U/μL), 2 μL Exo III (100 U/μL), 2 μL 10 × Exo I buffer, 2 μL 10 × NEBuffer™ 1 and 2 μL H_2O into the ligation product. The mixture was incubated first at 37 °C for 40 min, then at 80 °C for 20 min to inactivate the enzymes. After this step, the circular DNA template obtained was stored at 4 °C until use.

Competitive molecular complexation of aptamer-RBD and aptamer-cDNA

In order to confirm the feasibility of the protein assays, we need to verify the complexation of aptamer with cDNA and with S protein RBD, and the competitive nature of the molecular bindings. Firstly, we verified the complexation of FAM-labelled cDNA with biotin-tagged aptamer immobilized on streptavidin-coated MBs. The FAM-cDNA has a fully complementary sequence that matches the aptamer, while the FAM-ssDNA contains three mismatched bases. We used these two sequences at three different concentrations

(100 nM, 1 μM, 5 μM). As can be seen in Fig. S1, for all the cDNA concentrations tested, the cDNA led to lower fluorescence intensity in the supernatant than the mismatched ssDNA, confirming that the base-matched cDNA binds more effectively to the aptamer on the MBs.

To confirm that the aptamer is able to bind the S protein RBD, we tested the binding of MBs-supported cDNA with FAM-labelled aptamer (at two different concentrations (1 μM and 100 nM)) in the presence of S protein RBD (10 ng/mL, 100 ng/mL, 1 μg/mL,10 μg/mL). After removing the particles by magnetic separation, the fluorescence intensity of the supernatant was measured. With the concentration of RBD increased, the fluorescence intensity of the supernatant increased as well (Fig. S2). This result indicates that complexation with S protein RBD caused the FAM-labelled aptamer to dissociate from the cDNA on the MBs. The capability of S protein RBD to induce separation of FAM-aptamer from MBs-cDNA was investigated in more detail. As shown in Fig. S3, with the concentration of S-protein RBD increased, more FAM-aptamer molecules bound the RBD and dissociated from MBs-cDNA, making the fluorescence intensity of the liquid phase increased. Fig. S4 shows the result of electrophoresis of mixtures composed of different oligonucleotides and RBD. From the DNA bands we can see clearly that the sample containing cDNA and aptamer shows a single, slower-moving complex due to the hybridization of the aptamer and cDNA.

Optimization of assay conditions

After verification of the fluorometric and colorimetric systems for RBD detection, we proceeded to optimize the experimental conditions for the protein assays. The parameters that were optimized include the temperature used in the DNA hybridization, the reaction time of the RCA, the incubation time for reading the RCA products with the F-Q probe, and the substrate concentration in the colorimetric reaction catalyzed by the HRP-loaded DFs.

Optimization of RCA-enabled fluorometric system

The ratio of padlock DNA: primer is important to synthesize circular DNA template for subsequent rolling circle amplification. To optimize the synthesis condition, the volume of primer solution (10 μM) and padlock DNA solution (10 μM) was varied before T-4 DNA ligase was added to form the circular DNA. In Fig. S5, the gel image shows that when the padlock DNA: primer ratio changed from 2:1 to 1:2, the circular DNA band became the darkest accompanied by disappearance of the padlock DNA band. The other DNA bands were less affected. Therefore, we choose the padlock DNA: primer ratio of 1: 2 to prepare the circular DNA template in the remaining experiments.

The impact of reaction time of RCA was also studied. Samples were taken from the RCA reaction mixture at 0, 2 and 20 h and analyzed by gel

electrophoresis. As shown in Fig. S6, before RCA the reaction mixture showed multiple DNA bands of varying lengths. After 2 h of RCA, the product generated one intense band on the top of the gel. The 20 h RCA produced predominantly long strand DNA that appeared as a dark band on the top of the gel. Based on these results, we conclude that the designed circular DNA template and the RCA reaction were successful.

To use fluorescence emission for signal readout, we designed a dsDNA probe composed of a BHQ1-labelled strand and a slightly longer, FAM-labelled strand. The FAM-labelled strand is fully complementary to the cDNA/primer, therefore is able to hybridize with the cDNA/primer sequence and separate from the BHQ1-labelled strand, leading to fluorescence emission. As the RCA reaction produces long ssDNA having numerous copies of the cDNA sequence, the RCA reaction will amplify greatly the analytical signal (Fig. S7). Without RCA, the fluorescence signal caused by RBD-aptamer complexation is very weak (Fig. S7b). By adding the RCA step, the fluorescence signal caused by the RBD-aptamer recognition becomes significantly enhanced (Fig. S7c). Comparison of Fig. S7b with S7c shows clearly that the RCA is essential to achieve reliable detection of low concentration RBD (Fig. S7d).

To find out the optimal temperature for hybridization between aptamer and cDNA on MBs, we mixed FAM-labelled aptamer with MBs-cDNA at different temperatures. After magnetic separation, we measured the fluorescence intensity of the supernatant. The reduction of fluorescence intensity was used to evaluate the efficiency of the hybridization (Fig. S8). Normally, a temperature suitable for DNA hybridization is considered to be about 20-25 °C lower than the melting temperature (Tm). In our present system, the Tm of aptamer and cDNA is 80 °C and 40 °C, respectively. Therefore, we tested the hybridization at three different temperatures: 20 °C, 30 °C and 40 °C. As shown in Fig. S8, hybridization at 20 °C resulted in the highest uptake of FAM-aptamer by the cDNA on the MBs. Due to that the RCA time determines the length of the product DNA, it affects directly the intensity of fluorescence after adding the F-Q probe. We therefore studied the effect of carrying out the RCA reaction for 30 min, 60 min, 90 min, 120 min and 180 min. The RCA reaction was stopped by heating at 65 °C for 10 min. After adding the F-Q probe and incubation, we measured the fluorescence intensity of the mixture. As shown in Fig. S9, the fluorescence intensity increased when the reaction time increased from 30 min to 120 min. After 120 min the fluorescence intensity did not increase further. Therefore, we selected 120 min as the optimal RCA reaction time.

For fluorescence signal readout, the incubation time after addition of the F-Q probe is also important, because the strand separation of the probe and the

hybridization of the FAM-labelled strand with the RCA product is under both thermodynamic and kinetic control. To find out the suitable incubation time, we mixed the RCA products with the F-Q probe for 30 to 120 minutes and measured the fluorescence intensity. As shown in Fig. S10, after 45 minutes incubation the fluorescence intensity did not increase further. Therefore, in the remaining experiments the incubation time was fixed at 45 min.

Optimization of RCA-enabled colorimetric system

For colorimetric detection using the HRP-catalyzed reaction, the concentration of H_2O_2 is an important parameter for achieving reliable results. If the concentration of H_2O_2 is too high, it may lead to background oxidation. On the other hand, a too low concentration of H_2O_2 will not be able to convert TMB into oxidized form to generate the visible signal. Fig. S11a shows that with the H_2O_2 concentration increased from 0.05 mM to 0.5 mM, the absorbance value increased in proportion. When the H_2O_2 concentration increased from 0.25 mM to 0.5 mM, the absorbance value remains almost unchanged. Therefore, we choose 0.25 mM as the optimal concentration of H_2O_2 in all subsequent experiments.

The reaction time of the colorimetric system was varied to find out the most suitable condition. The HRP-catalyzed reaction was carried out for different periods before the reaction was stopped. In Fig. S11b, it shows that from 4 to 10 min, the absorbance value continued to increase. After 10 min reaction, the absorbance did not increase any further. Therefore, we chose 10 min as the reaction time in the final colorimetric assay.

Assay protocols for determination of RBD

Fluorometric assay

1. Incubate sample (100 μL) with 50 μL of MBs-cDNA/apt suspension for 1 h.

2. Separate and wash the MBs 3 times with 200 μL 1 × PBS buffer. Re-suspend the MBs in 30 μL 1 × PBS buffer to give MBs-cDNA to be used in RCA.

3. Add circular DNA template (2 μL) to the MBs-cDNA (4 μL) and mix with 1 μL recombinant albumin, 1 μL phi29 DNA polymerase (10 U/μL), 4 μL dNTPs (10 mM), 3 μL 10 × phi29 DNA polymerase buffer and 15 μL H_2O. Incubate the mixture with gentle agitation at 30 °C for 2 h. Heat the mixture at 65 °C for 10 min to inactivate the enzyme.

4. Add 10 μL of F-Q probe and 60 μL H_2O. Incubate the mixture for 45 min.

5. Measure fluorescence intensity of the mixture (λ_{ex} = 488 nm, (λ_{ex} = 520 nm).

Colorimetric assay

1. Incubate sample (100 μL) with 50 μL of MBs-cDNA/apt suspension for 1 h.

2. Separate and wash the MBs 3 times with 200 μL 1 × PBS buffer. Re-suspend the MBs in 30 μL 1 × PBS buffer to give MBs-cDNA to be used in RCA.

3. Add circular DNA template (2 μL) to the MBs-cDNA (4 μL) and mix with 1 μL recombinant albumin, 1 μL phi29 DNA polymerase (10 U/μL), 4 μL dNTPs (10 mM), 3 μL 10 × phi29 DNA polymerase buffer, 15 μL H_2O and 5 μL HRP solution (1 mg/mL). Incubate the mixture with gentle agitation at 30 °C for 20 h. Heat the mixture at 65 °C for 10 min to inactivate the enzyme.

4. Separate and wash the DNA flower with 100 μL water, resuspend the DNA flower in 30 μL water.

5. Mix the DNA flower (10 μL) with 80 μL acetate buffer (pH 5, 0.1 M). Add 5 μL 35% H_2O_2 and 5 μL TMB (0.025 mM). Incubate the mixture for 10 min. Add 50 μL of stop solution (0.2 M H_2SO_4) to quench the reaction. Remove the magnetic particles.

6. Measure absorbance of the supernatant at 450 nm.

Table S1 Oligonucleotide sequences used in this study.

Oligonucleotide	Sequence (5’-3’)
Aptamer	CAGCACCGACCTTGTGCTTTGGGAGTGCTGGTCCAAGGGCG TTAATGGACA
Biotin-Aptamer	Biotin-CAGCACCGACCTTGTGCTTTGGGAGTGCTGGTCCAAGGGCG TTAATGGACA
FAM-Aptamer	(FAM)-CAGCACCGACCTTGTGCTTTGGGAGTGCTGGTCCAAGGGCG TTAATGGACA
Primer (cDNA)	TGTCCATTAACGCCCT
Biotin-ssDNA	Biotin-TGTCCATTAACGCCCT
FAM-cDNA	(FAM)-TGTCCATTAACGCCCT
FAM-ssDNA	(FAM)-TGTCCAAATACGCCCT
Padlock DNA	P-AATGGACATATGTAGGACAAGATTGTAGGTCAGAACTCAGGTGCAAG AAACTGTGAAGATCGGGAA AGGGCGTT
Probe F	(FAM)-AGGGCGTTAA TGGACA
Probe Q	TTAACGCCCT-(BHQ1)

Table S2 Buffer composition used in this study.

Buffer	Composition
T-4 DNA buffer (1×)	50 mM Tris-HCl, 10 mM $MgCl_2$, 1 mM ATP and 10 mM DTT, pH 7.5
NEBuffer 1 (1×)	10 mM Bis-Tris-Propane-HCl, 10 mM $MgCl_2$, 1 mM DTT, pH 7
PBS buffer (1×)	137 mM NaCl, 2.7 mM KCl, 10 mM Na_2HPO_4, 1.8 mM KH_2PO_4
Binding buffer	10 mM Tris, 0.5 M NaCl, 1 mM EDTA, pH 7.4
Exo I buffer (1×)	67 mM Glycine-KOH,6.7 mM $MgCl_2$, 10 mM β-ME, pH 9.5
Phi29 DNA polymerase buffer (1×)	50 mM Tris-HCl, 10 mM $MgCl_2$, 10 mM $(NH_4)_2SO_4$ and 4 mM DTT, pH 7.5
TBE buffer (1×)	90 mM Tris-HCl, 2 mM EDTA and 90 mM boric acid, pH 7.8
Tris-EDTA buffer	10 mM Tris-HCl, 1 mM EDTA, pH 8
Tris-HCl buffer	10 mM Tris-HCl, pH 7.5
Recombinant albumin buffer	20 mM Tris-HCl, 100 mM KCl, 0.1 mM EDTA, 50% glycerol, pH 8

Table S3 Comparison of analytical performance for detection of SARS-CoV-2 spike protein.

Method/sensor	Concentration range	Detection limit	Reference
Shrinky-Dink© electrode	-	1 ag/mL	[3]
Surface-enhanced Raman spectroscopy	-	10^{-9} M	[4]
Carbon nanotube-based, near-infrared nanosensor	-	12.6 nM	[5]
Surface plasmon resonance aptasensor	1 nM–100 nM	0.26 nM	[6]
Graphene-impedimetric immunosensor	-	2.4 ng/mL	[7]
Immunosensor	0.0012–120 pg/mL	0.58 fg/mL	[8]
Optical biosensing	12.5–400 pM	12.5 pM	[9]
Fluorescence detection	0.001 - 100 ng/mL	0.11 pg/mL	This work
Colorimetric detection	0.001 – 100 ng/mL	0.904 pg/mL	This work

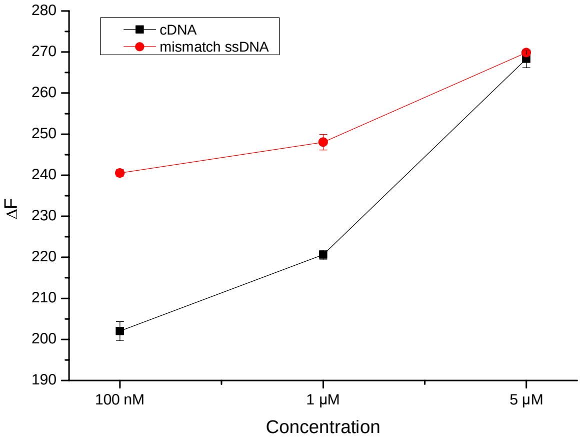

Fig. S1 Verification of molecular complexation of aptamer with cDNA. In this experiment, 25 μL of two DNA sequences (FAM-cDNA and FAM-mismatched ssDNA) at three different concentrations (100 nM, 1 μM, 5 μM) were mixed with 50 μL of MBs-aptamer (1 μM) and 125 μL 1× PBS buffer. The mixture was incubated at room temperature for 1 h. After magnetic separation, the fluorescence intensity of the supernatant was measured.

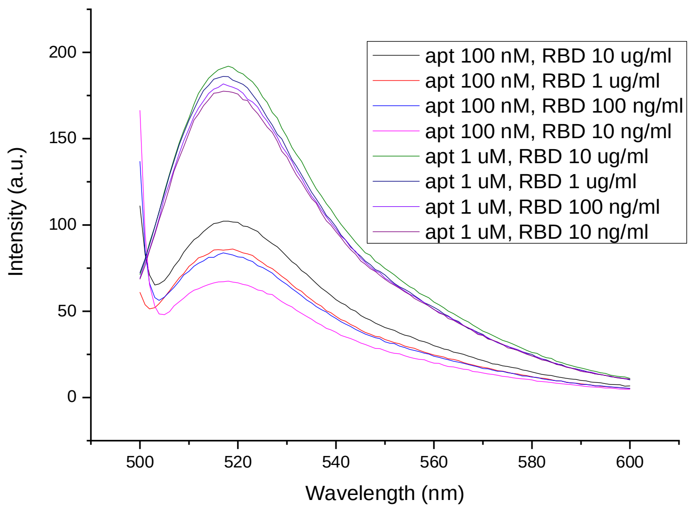

Fig. S2 Dissociation of FAM-aptamer from MBs-cDNA caused by different concentrations of S protein RBD. In this experiment, 25 μL MBs-cDNA (1 μM) was mixed with 50 μL FAM-aptamer solution (1 μM or 100 nM) and 125 μL 1× PBS buffer. After incubation at room temperature for 1 h, 100 μL of different concentrations of RBD protein (10 ng mL^{-1}, 100 ng mL^{-1}, 1 μg mL^{-1}, 10 μg mL^{-1}) was added, and the obtained mixture was incubated on a rocking table at room temperature for 60 min. After magnetic separation, the fluorescence intensity of the supernatant was measured.

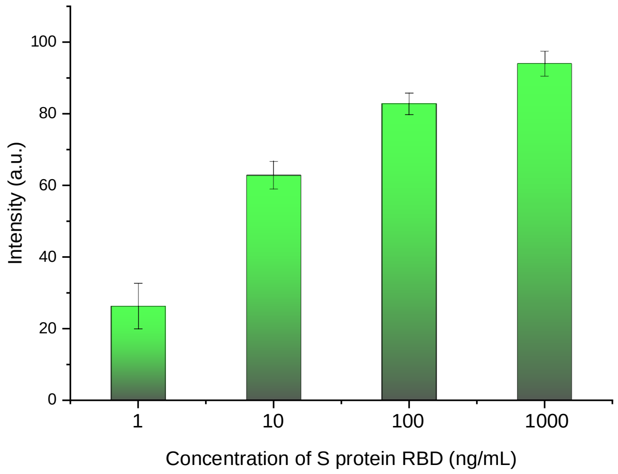

Fig. S3 Dissociation of FAM-aptamer from MBs-cDNA caused by different concentrations of S protein RBD. In this experiment, 25 μL MBs-cDNA (1 μM) and 50 μL FAM-aptamer solution (100 nM) were mixed in 1× PBS buffer at room temperature for 1 h. After addition of 100 μL different concentrations of S protein RBD (1 ng mL^{-1}, 10 ng mL^{-1}, 100 ng mL^{-1}, 1 μg mL^{-1}), the mixture was incubated at room temperature for 60 min. After magnetic separation, the fluorescence intensity of the supernatant was measured.

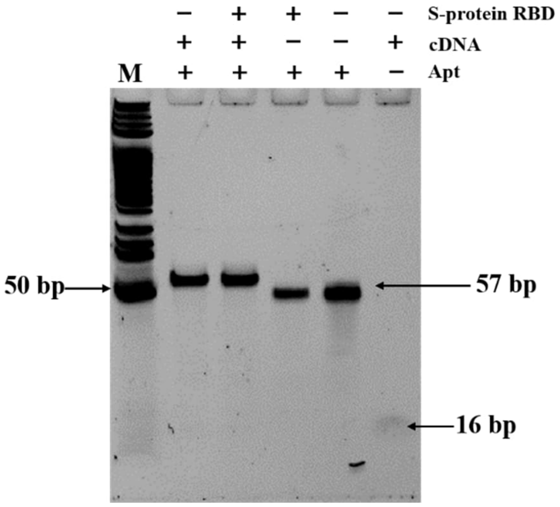

Fig. S4 Gel electrophoresis (12% PAGE) of DNA samples with and without S protein RBD. M: 50 bp marker; Apt: aptamer (1 μM); cDNA (1 μM); S protein RBD (10 ng mL^{-1}). In this experiment, 50 μL aptamer (1 μM), 50 μL cDNA (1 μM) and 100 μL RBD in 1× PBS buffer (10 ng mL^{-1}) were used. The three solutions were combined and topped with 1× PBS buffer to give 200 μL of five different samples. Sample 1: 50 μL aptamer, 50 μL cDNA and 100 μL 1× PBS buffer; Sample 2: 50 μL aptamer, 50 μL cDNA and 100 μL RBD; Sample 3: 50 μL aptamer, 100 μL RBD and 50 μL 1× PBS buffer; Sample 4: 50 μL aptamer and 150 μL 1× PBS buffer; Sample 5: 50 μL cDNA and 150 μL 1× PBS buffer. The samples were incubated at room temperature for 1 h. From each sample, 10 μL was loaded to the 12% polyacrylamide gel.

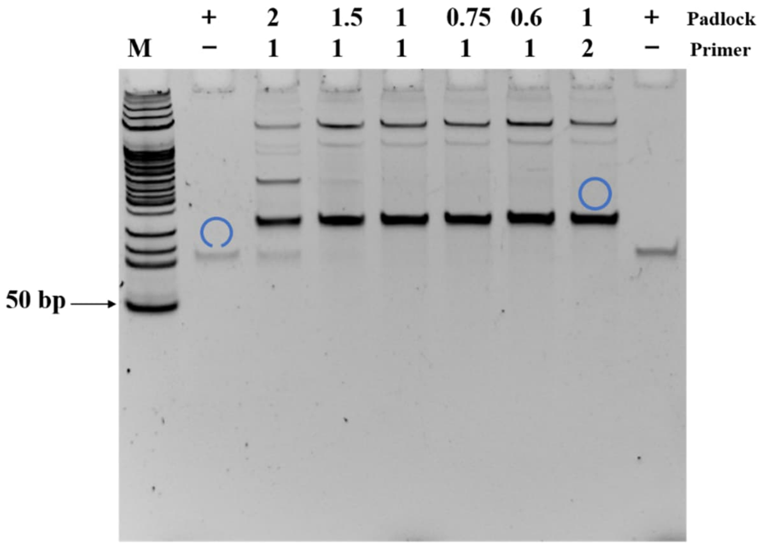

Fig. S5 Optimization of padlock DNA: primer ratio for synthesis of circular DNA template. In this experiment, different volumes of 10 μM primer solution (0 μL, 1 μL, 2 μL, 3 μL, 4 μL, 5 μL and 6 μL) was mixed with 3 μL of padlock DNA (10 μM) and 5 μL 10× T-4 DNA ligase buffer. The final volume was adjusted to 49 μL by adding purified H_2O. The mixture was incubated at 95 °C for 5 min, then cooled to room temperature. Finally, T-4 DNA ligase (1 μL) was added and the obtained mixture was gently stirred at room temperature for 2 h. After the ligation reaction, 10 μL of each sample was loaded to the 12% polyacrylamide gel for electrophoresis.

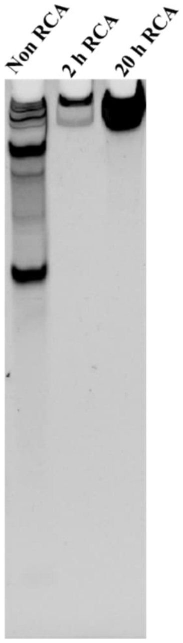

Fig. S6 Gel electrophoresis of RCA products. The RCA reaction was performed at 30 °C using 2 μL circular DNA template and 4 μL of 1 μM primer. Reaction mixture of RCA (10 μL) was loaded to 12% polyacrylamide gel.

(a)

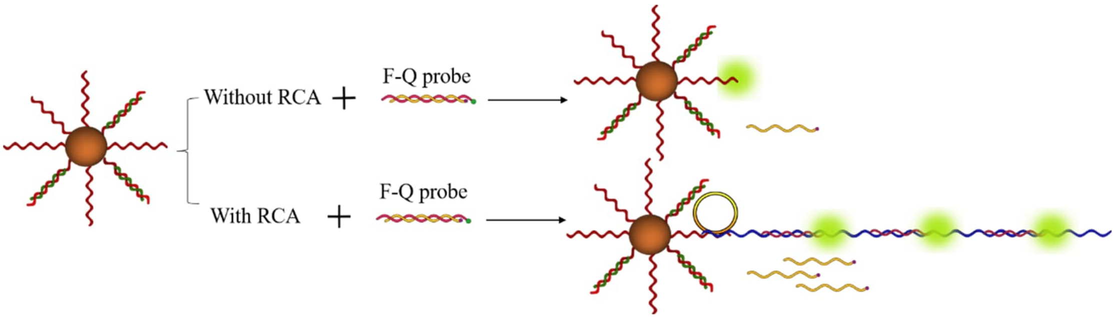

(b)

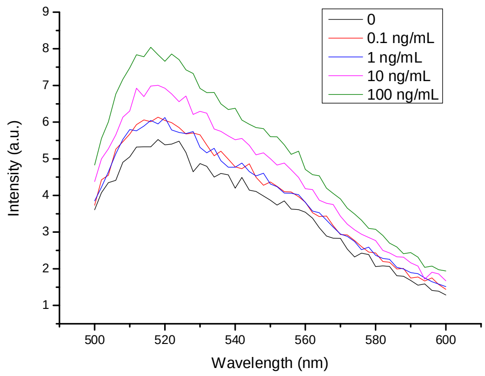

(c)

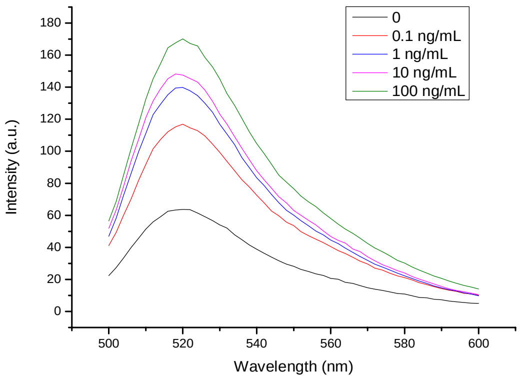

(d)

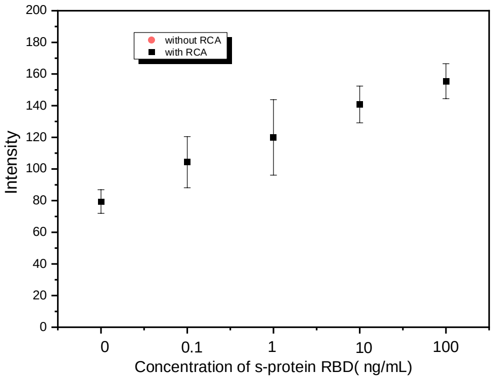

Fig. S7 Verification of RCA-enabled fluorometric detection of RBD. (a) Principle of fluorometric detection of RBD enabled by RCA. (b) Fluorescence signal of RBD assay without RCA. (c) Fluorescence signal of RBD assay using RCA. (d) Comparison of signal intensities in RBD assays with and without RCA.

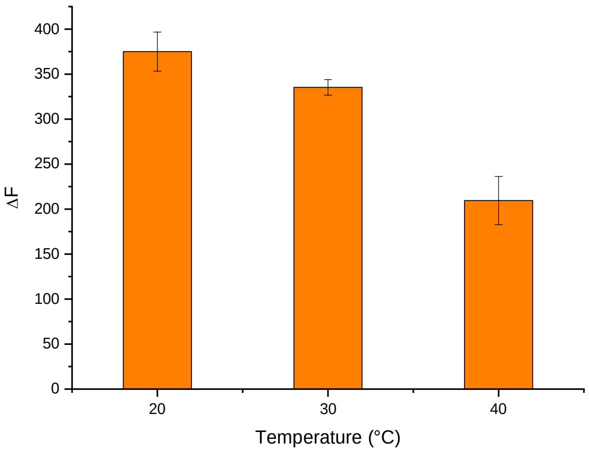

Fig. S8 Optimization of hybridization temperature. FAM-Apt (10 μM, 50 μL) was heated at 80 °C for 3 min. Biotin-cDNA (10 μM, 25 μL) was heated at 40 °C for 10 min. After cooling to room temperature, the two solutions were mixed with 125 μL 1× PBS buffer and incubated at different temperatures (20 °C, 30 °C and 40 °C) for 1 h. The solution was then added to 200 μL MBs, and incubated at room temperature for 1 h. The MBs-cDNA/apt was collected by magnetic separation, and washed with 1× PBS buffer 3 times. Finally, the MBs-cDNA/apt was re-suspended in 200 μL 1× PBS buffer to measure the fluorescence intensity.

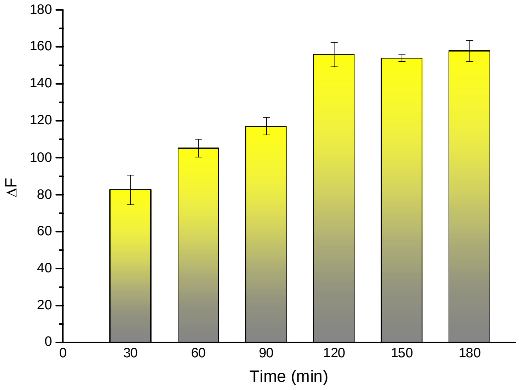

Fig. S9 Optimization of the amplification reaction time for fluorescence probe detection. The S protein RBD concentration was 10 ng mL^{-1}. The time of incubation of RCA product with F-Q probe was 45 min. The RCA reaction was carried out at 30 °C for different time before the polymerase was inactivated.

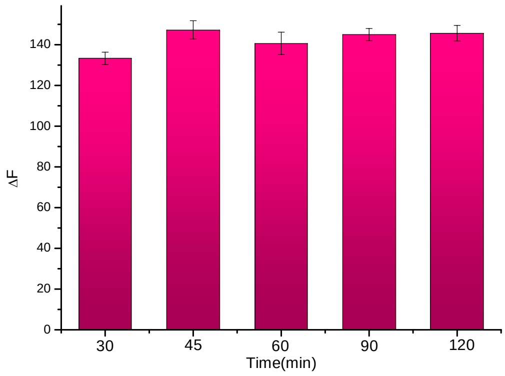

Fig. S10 Optimization of time of hybridization between RCA product and F-Q probe. The RCA reaction continued for 120 min before the polymerase was inactivated by heating at 65 °C for 10 min. The RCA product was mixed with F-Q probe at room temperature for different time before the fluorescence signal was measured. Ex: 488 nm; Em: 520 nm.

(a) (b)

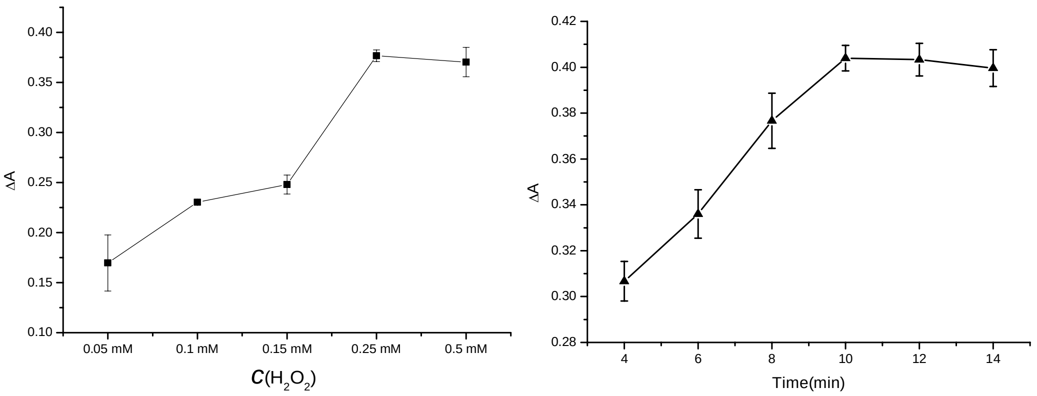

Fig. S11 Optimization of calorimetric reaction conditions. (a) Optimization of H_2O_2 concentration. The TMB concentration was 0.025 mM, and the reaction time was 10 min. (b) Optimization of reaction time. The concentration of H_2O_2 was 0.25 mM and the TMB concentration was 0.025 mM. All the experiments were carried out using RBD concentration at 1 ng mL^{-1}.

References

1. Modh H, Scheper T, Walter JG (2018) Aptamer-Modified Magnetic Beads in Biosensing. Sensors (Basel) 18(4):1041. https://doi.org/10.3390/s18041041
2. Liu D, Daubendiek SL, Zillman MA, Ryan K, Kool ET (1996) Rolling Circle DNA Synthesis: Small Circular Oligonucleotides as Efficient Templates for DNA Polymerases. J Am Chem Soc 118(7):1587-1594. https://doi.org/10.1021/ja952786k
3. Zakashansky JA, Imamura AH, Salgado DF, Mercieca HCR, Aguas RF, Lao AM, Khine M (2021) Detection of the SARS-CoV-2 spike protein in saliva with Shrinky-Dink© electrodes. Anal Methods, *13*(7), 874-883. https://doi.org/10.1039/D1AY00041A
4. Abdullah MB, Dab C, Almalki M, Alnaim A, Abuzir A, Awada C (2022) Ultrafast Detection of SARS-COV-2 Spike protein (S) and receptor-binding domain (RBD) in Saliva using Surface-enhanced raman spectroscopy. Appl Sci, 12(10), 5039. https://doi.org/10.3390/app12105039
5. Pinals RL, Ledesma F, Yang D, Navarro N, Jeong S, Pak JE, Landry MP (2021) Rapid SARS-CoV-2 spike protein detection by carbon nanotube-based near-infrared nanosensors. Nano Lett, 21(5), 2272-2280. https://doi.org/10.1021/acs.nanolett.1c00118
6. Lewis T, Giroux E, Jovic M, Martic-Milne S (2021) Localized surface plasmon resonance aptasensor for selective detection of SARS-CoV-2 S1 protein. Analyst, 146(23), 7207-7217. https://doi.org/10.1039/D1AN01458G
7. Zaccariotto GC, Silva MK, Rocha GS, Cesarino I (2021) A novel method for the detection of SARS-CoV-2 based on graphene-impedimetric immunosensor. Materials, 14(15), 4230. https://doi.org/10.3390/ma14154230
8. Aydın EB, Aydın M, Sezginturk MK (2021) New impedimetric sandwich immunosensor for ultrasensitive and highly specific detection of spike receptor binding domain protein of SARS-CoV-2. ACS Biomater Sci Eng, 7(8), 3874-3885. https://doi.org/10.1021/acsbiomaterials.1c00580
9. Tao Y, Bian S, Wang P, Zhang H, Bi W, Zhu P, Sawan M (2022) Rapid Optical Biosensing of SARS-CoV-2 Spike Proteins in Artificial Samples. Sensors, 22(10), 3768. https://doi.org/10.3390/s22103768
